# Metabolic Disorder, Inflammation, and Deregulated Molecular Pathways Converging in Pancreatic Cancer Development: Implications for New Therapeutic Strategies

**DOI:** 10.3390/cancers3010446

**Published:** 2011-01-24

**Authors:** Yoshiharu Motoo, Takeo Shimasaki, Yasuhito Ishigaki, Hideo Nakajima, Kazuyuki Kawakami, Toshinari Minamoto

**Affiliations:** 1 Department of Medical Oncology, Kanazawa Medical University, 1-1 Daigaku, Uchinada, Ishikawa 920-0293, Japan; E-Mails: takeo@kanazawa-med.ac.jp (T.S.); hideonak@kanazawa-med.ac.jp (H.N.); 2 Medical Research Institute, Kanazawa Medical University, 1-1 Daigaku, Uchinada, Ishikawa 920-0293, Japan; E-Mail: ishigaki@kanazawa-med.ac.jp; 3 Division of Translational & Clinical Oncology, Cancer Research Institute, Kanazawa University, Kanazawa, Japan; E-Mails: kawakami@med.kanazawa-u.ac.jp (K.K.); minamoto@staff.kanazawa-u.ac.jp (T.M.)

**Keywords:** pancreatic cancer, glucose intolerance, inflammation, oncogenic pathways, therapeutic target, GSK3β

## Abstract

Pancreatic cancer develops and progresses through complex, cumulative biological processes involving metabolic disorder, local inflammation, and deregulated molecular pathways. The resulting tumor aggressiveness hampers surgical intervention and renders pancreatic cancer resistant to standard chemotherapy and radiation therapy. Based on these pathologic properties, several therapeutic strategies are being developed to reverse refractory pancreatic cancer. Here, we outline molecular targeting therapies, which are primarily directed against growth factor receptor-type tyrosine kinases deregulated in tumors, but have failed to improve the survival of pancreatic cancer patients. Glycogen synthase kinase-3β (GSK3β) is a member of a serine/threonine protein kinase family that plays a critical role in various cellular pathways. GSK3β has also emerged as a mediator of pathological states, including glucose intolerance, inflammation, and various cancers (e.g., pancreatic cancer). We review recent studies that demonstrate the anti-tumor effects of GSK3β inhibition alone or in combination with chemotherapy and radiation. GSK3β inhibition may exert indirect anti-tumor actions in pancreatic cancer by modulating metabolic disorder and inflammation.

## 1. Introduction

Pancreatic cancer is a devastating disease and a major unresolved health problem due to its late clinical diagnosis and predisposition towards metastasis [1,2]. Pancreatic cancer is characterized by highly proliferative and invasive tumor cells [3]. Despite progress in approaches to treatment [4], such aggressive biological behavior thwarts early diagnosis and curative surgical intervention and renders tumors resistant to conventional chemotherapy, radiation therapy, and their combination [5-7], leading to a poor prognosis [8]. Therefore, understanding the detailed molecular and biological basis of pancreatic cancer pathogenesis facilitates advances in the diagnosis, treatment, and prevention of this disease.

**Figure 1.**
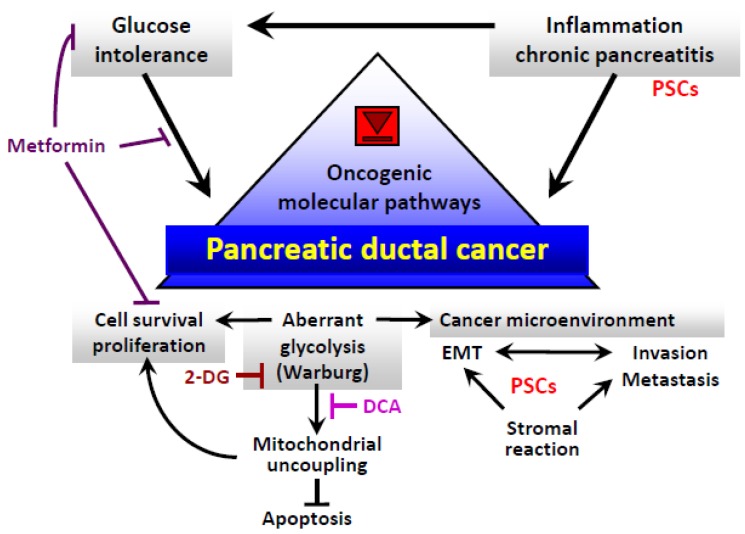
Molecular and biological pathways involved in the development and progression of pancreatic cancer and putative mechanisms underlying the anticancer effects of metformin, 2-deoxyglucose (2-DG), and dichloroacetate (DCA). The gray triangle in a box indicates a molecular target. Abbreviations: EMT, epithelial-to-mesenchymal transition; PSCs, pancreatic stellate cells; Warburg, Warburg effect.

Pancreatic cancer develops through a series of complex, cumulative biological processes involving metabolic disorder and chronic local inflammation in association with stromal changes and deregulated molecular pathways (Figure 1). Based on these pathologic properties, new therapeutic strategies are being developed to reverse the refractory stage of pancreatic cancer. Here, we review the multidimensional processes leading to pancreatic cancer development and progression, and discuss biology-based therapeutic alternatives to conventional cancer treatments. In addition, we highlight an emerging strategy for cancer treatment that targets glycogen synthase kinase 3β (GSK3β), focusing on the effect of its deregulation on pancreatic cancer.

## 2. Metabolic Disorder Coincides with Pancreatic Cancer

Although systemic metabolic disorders such as obesity and type II diabetes mellitus (DM) influence the risk of developing pancreatic cancer and clinical outcomes (reviewed in [9,10]), the abnormal metabolic profile of cancer cells dictates their survival, proliferation, and invasion, as well as susceptibility to chemotherapy and radiation [11,12]. Thus, metabolic disorder and altered tumor cell metabolism are potential targets for cancer treatment and (chemo) prevention [13,14].

### 2.1. Obesity, Glucose Intolerance, and Pancreatic Cancer

The association between obesity/DM and pancreatic cancer risk has long been controversial. However, recent reviews and meta-analyses of prospective observational studies have demonstrated that obesity, defined by an increased body mass index, is significantly associated with a risk of pancreatic cancer development [15,16]. DM is also a clinical manifestation of pancreatic cancer, and case-control and prospective studies have demonstrated an increased risk of pancreatic cancer in patients with long-term DM [17-19]. Obesity is associated with the early manifestations of pancreatic cancer and lower overall patient survival [20], although the influence of DM on pancreatic cancer progression or morbidity is not clear [10]. Possible mechanisms behind the association between obesity and worse clinical outcomes may include increased risk of DM, thrombosis, or other comorbidities; impaired immune response leading to aggressive tumor behavior; and poor response to conventional anticancer therapies [10]. The effect of obesity and DM on the development and progression of pancreatic cancer appears to be mediated by adipokines, reactive oxygen species (ROS), inflammatory cytokines, and insulin resistance, which result in activation of insulin-like growth factor-1 (IGF-1) and mammalian target of rapamycin (mTOR) pathways (reviewed in [10]).

A number of studies have reported the potential role of glucose-lowering therapies in reducing the risk of pancreatic cancer (reviewed in [21]). Metformin is a biguanide that is most frequently prescribed for diabetes [22]. Recently, a comprehensive review and meta-analysis of epidemiologic studies demonstrated an inverse correlation between the use of metformin and incidence of pancreatic cancer and overall survival of patients with diabetes [23]. Increasing evidence also suggests that metformin exerts a therapeutic effect against cancer [24,25]. Metformin decreases insulin resistance and indirectly reduces levels of insulin and IGF-1, which promote cancer cell proliferation [26]. Further, metformin activates the tumor suppressor pathway mediated by liver kinase B1 (LKB1) and 5′ AMP-activated protein kinase (AMPK), an important sensor of cellular energy status [27], thereby inactivating mTOR signaling [14]. IGF-1 receptor and G protein-coupled receptor signaling is implicated in the autocrine-paracrine stimulation of a variety of malignant tumors, including exocrine pancreatic cancer. Recent studies demonstrated that metformin-induced activation of AMPK disrupts the crosstalk between insulin/IGF-1 receptor and G protein-coupled receptor signaling pathways in pancreatic cancer cells and inhibits proliferation of these cells in xenograft models, suggesting this crosstalk as a target for treatment of pancreatic cancer by metformin [28].

### 2.2. Distinct Metabolic Properties of Cancer Cells

The fundamental metabolic characteristics of cancer cells include increased glucose uptake, aerobic glycolysis even under normoxic condition (Warburg effect) [29], and impaired oxidative phosphorylation in the tricarboxylic acid (TCA) cycle, which results in mitochondrial uncoupling [30]. These properties could explain the ability of cancer cells to survive, invade host tissues, and resist the induction of apoptosis by chemotherapeutic agents and ionizing radiation [30,31]. However, the glycolytic phenotype of cancer cells is a potential target for cancer diagnosis and treatment [32]. For example, enhanced glucose uptake by cancer cells can be used to visualize cancer by positron emission tomography (PET) using the radioisotope-labeled glucose analogue 2-[^18^F]-fluoro-2-deoxy-D-glucose (FDG). FDG-PET in combination with computed tomography (PET-CT) enables detection of metastatic lesions of most cancers with both sensitivity and specificity greater than 90% [33]. Pharmacologic agents targeting the glycolytic phenotype of cancer cells include 2-deoxy-D-glucose (2-DG) and dichloroacetate (DCA).

A glucose analogue, 2-DG, is the most attractive agent for targeting aberrant glucose metabolism in cancer cells [34]. 2-DG inhibits glucose transport by competing with glucose transporters and is subsequently phosphorylated by hexokinase to form 2-DG-6-phospate. Phosphorylated 2-DG is not further metabolized by inhibiting glucose-6-phosphate isomerase, thereby reducing the production of adenosine triphosphate (ATP) and nicotinamide adenine dinucleotide phosphate (NADPH) from glycolysis coupled with the pentose phosphate pathway. Thus, 2-DG exerts antitumor effects by starving cancer cells [34].

The association between the glycolytic phenotype (*i.e.*, TCA cycle defects) and resistance to apoptosis is attributed to decreased mitochondrial hydrogen peroxide production and cytochrome C release [30,31]. Pyruvate dehydrogenase (PDH) plays a crucial role in triggering the TCA cycle by converting pyruvate to citric acid. PDH kinase 1 (PDK1), which phosphorylates and inactivates PDH, is frequently overactivated in cancer cells, resulting in an impaired TCA cycle and mitochondrial hyperpolarization. Thus, inhibiting PDK1 would re-activate PDH and restore mitochondrial membrane polarity, thereby facilitating cancer cell apoptosis in response to chemotherapeutic agents and radiation. DCA, an orally bio-available small molecule, is a well characterized PDK1 inhibitor. The ability of DCA to inhibit lactate production (by stimulating PDH and the TCA cycle) has been long used to treat lactic acidosis, which complicates inherited mitochondrial disorders [35,36]. A recent study demonstrated that DCA induces cancer cell apoptosis by selectively inhibiting PDK1 in cancer cells, leading to metabolic remodeling from glycolysis to glucose oxidation and normalization of mitochondrial function [37]. A recent clinical trial of oral DCA in children with congenital lactic acidosis reported that DCA was well tolerated and safe [36]. Thus, orally available DCA is a promising selective anticancer agent.

## 3. Inflammation and Stromal Reactions in Pancreatic Cancer

The well recognized link between chronic inflammation and tumor development in many organs [38] is consistent with the reported causative association and interaction between chronic pancreatitis and pancreatic cancer [39-41]. In addition to the etiologic role of inflammation in carcinogenesis, systemic and local inflammation are frequent manifestations of pancreatic cancer and have been implicated in tumor progression and clinical outcomes [39].

### 3.1. Chronic Pancreatitis and Pancreatic Cancer

Chronic pancreatitis is a risk factor for developing pancreatic cancer [39-41]. This association is supported by a recent meta-analysis of 22 well-performed epidemiologic studies [42]. The risk of developing pancreatic cancer for patients with hereditary pancreatitis is much higher than for patients with sporadic chronic pancreatitis [43-45]. The incidence of chronic pancreatitis in the general population is only about 5 to 10 per 100,000 persons a year, as estimated from hospitalization data. In particular, hereditary pancreatitis caused by germline mutations in the cationic trypsinogen gene [46] accounts for less than 1% of all chronic pancreatitis cases. Therefore, chronic pancreatitis does not constitute a main precursor of pancreatic cancer [9,42].

Despite this low incidence, both forms of chronic pancreatitis have provided substantial evidence for putative inflammatory mechanisms contributing to pancreatic cancer development and progression, including proinflammatory cytokines, nuclear factor-κB (NF-κB), cyclooxygenase-2 (COX-2), peroxisome proliferator-activated receptor-γ (PPARγ), nitric oxide (NO) synthesized by inducible NO synthase (iNOS), DNA damage caused by release of proteolytic enzymes and ROS, and somatic mutations in oncogenes (e.g., K-ras) and tumor suppressor genes (e.g., p53, p16, DPC4/Smad) [47-49]. Pancreatic cancer progression shares these molecular alterations, which are promising targets for early molecular diagnosis, treatment, and prevention of the disease [47-49].

Histopathologic findings of chronic pancreatitis include marked fibrosis, in which pancreatic stellate cells (PSCs) play a crucial role [50,51]. In the normal pancreas, quiescent PSCs produce vitamin A in the periacinar and interlobular space. In response to pancreatic inflammation, PSCs are activated and transformed to a myofibroblast-like phenotype; they proliferate, migrate and produce extracellular matrix components (e.g., collagens, laminin, fibronectin), matrix metalloproteinases, and tissue inhibitors of matrix metalloproteinases. This phenotypic change is induced by inflammatory cytokines (e.g., tumor necrosis factor, interleukin-1, and interleukin-6), growth factors such as transforming growth factor (TGF)-β1, TGF-α, platelet-derived growth factor (PDGF), and fibroblast growth factor (FGF)-2; and ROS [52]. In addition to their roles in the pathogenesis of chronic pancreatitis and cancer, these factors also promote pancreatic cancer progression [50,51].

### 3.2. Cancer-Stromal Interaction and Tumor Microenvironment

Chronic inflammation, desmoplastic stromal reaction, and neovascularization associated with pancreatic cancer combine to produce a distinct tissue microenvironment where cancer cell proliferation and invasion are facilitated by cancer-stromal interactions [53,54]. Activated PSCs are primarily responsible for the desmoplastic reaction and tumor angiogenesis in response to various growth factors such as TGF-β, FGF, HGF, and IGF-1. Results from *in vitro* and *in vivo* studies suggest that cancer cells recruit PSCs to tumors, where PSCs promote cancer cell proliferation and facilitate their invasion and metastasis, and that FGF and PDGF mediate these interactions between the two cell types (reviewed in [55]).

Phenotypic changes also occur in cancer cells, as represented by epithelial-mesenchymal transition (EMT) at the interface between tumor and stroma, in which epithelial cells undergo morphologic changes characterized by a transition from epithelial to fibroblastic (mesenchymal cell) phenotypes. Most factors involved in pancreatic cancer-stromal interactions have the potential to induce EMT in cancer cells. This process involves loss of cell-to-cell adhesion and E-cadherin expression, actin cytoskeleton reorganization, and increased expression of mesenchymal molecules (e.g., vimentin, fibronectin, α-smooth muscle actin, N-cadherin). In this way, EMT facilitates the invasion and metastasis of cancer cells and renders them resistant to chemotherapy and radiation [56,57]. Accordingly, growth factors such as TGF-β and hepatocyte growth factor (HGF, or its receptor c-Met) that are involved in cancer-stromal interactions and EMT have been well studied in order to develop therapeutic strategies targeting these factors [53,54].

## 4. Targeting Molecular Pathways Deregulated in Pancreatic Cancer

Most cases of pancreatic cancer are resistant to conventional chemotherapy and radiation therapy [5-7]; therefore, new strategies are needed to enhance the antitumor effects of gemcitabine, which is the standard chemotherapeutic agent used to treat pancreatic cancer [58]. These new classes of biology-based treatment modalities include molecular target-directed therapies.

### 4.1. Deregulated Molecular Pathways Mediated by Receptor-Type Tyrosine Kinases

**Figure 2.**
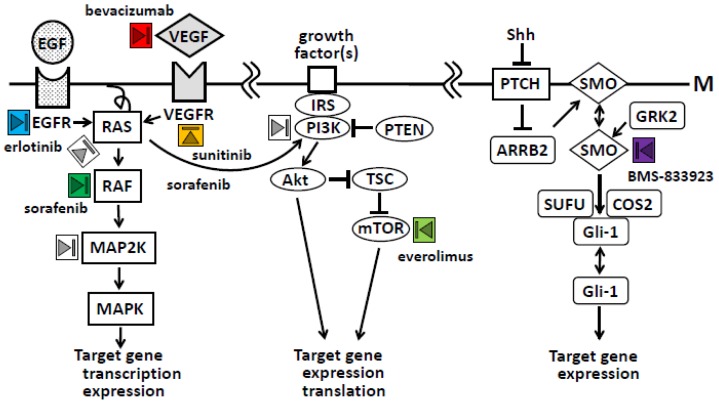
Critical molecular pathways leading to the development and progression of pancreatic cancer. Abbreviations: ARRB2, arrestin β2 ; COS2, kinesin-related protein Costal 2; DUSP6, dual specificity phosphatase 6; EGF, epidermal growth factor; EGFR, EGF receptor; GRK2, G protein-coupled receptor kinase-2; IRS, insulin receptor substrate 1; M, cell membrane; MAPK, mitogen-activated protein kinase; MAP2K, MAP kinase kinases; mTOR, mammalian target of rapamycin; PI3K, phosphatidylinositol 3-kinase; PTCH, patched; PTEN, phosphatase and tensin homolog deleted in chromosome 10; Shh, sonic hedgehog; SMO, smoothened; SUFU, suppressor of fused; TSC, tuberous sclerosis complex; VEGF, vascular endothelial growth factor; VEGFR, VEGF receptor. The gray triangle in a box indicates a target for drug development.

Molecular studies have investigated the complex genetic mechanisms of cancer, which involve multidirectional signal transduction pathways [3,59,60]. As shown in Figure 2, the major signal transduction pathways in pancreatic cancer pathogenesis and progression are RAS/mitogen-activated protein kinase (MAPK), phosphatidylinositol 3-kinase (PI3K)/Akt/mTOR, and hedgehog pathways [3]. The receptor tyrosine kinase family and their ligands, which include epidermal growth factor (EGF) receptor (EGFR), vascular endothelial growth factor (VEGF) receptor (VEGFR), and PDGF receptor (PDGFR), are targets of therapy because they are overexpressed in many tumor types, including pancreatic cancer [61].

### 4.2. Pharmacologic Agents that Target Deregulated Kinases

Currently available agents that target these factors include anti-EGFR antibodies (cetuximab, panitumumab), small molecule EGFR inhibitors (gefitinib, erlotinib), an anti-VEGF antibody (bevacizumab), and a small molecule VEGFR inhibitor (axitinib). A number of phase III clinical trials have tested kinase inhibitors as monotherapy or in combination therapy with gemcitabine for pancreatic cancer, but other than the combination of erlotinib and gemcitabine [62], these approaches have produced few therapeutic benefits [63]. Characterization of new molecular targets is necessary in order to develop strategies that enhance the effect of gemcitabine and improve the survival rate. Recent studies have pursued potential kinases as targets for new anticancer agents [64] and evaluated agents targeting known kinases (e.g., EGFR, check-point kinase 1) to combine with gemcitabine in order to improve its antitumor effects [65].

## 5. GSK3β, an Emerging Therapeutic Target in Cancer

GSK3β has emerged as a critical factor that plays distinct pathologic roles in glucose intolerance, inflammation, and in various cancer types (e.g., pancreatic cancer). Here, we briefly review recent studies, including our own, that demonstrate the direct anticancer effects of GSK3β inhibition, alone or in combination with chemotherapy and radiation.

### 5.1. Outline of GSK3β and its Involvement in Chronic Progressive Diseases

GSK3β was first identified as a serine/threonine protein kinase that regulates glucose/glycogen metabolism under the control of insulin signaling. Unlike most protein kinases, GSK3β is active in normal cells, and its activity is controlled by subcellular localization, differential phosphorylation at serine 9 (S9) and tyrosine 216 (Y216) residues, and different binding partners. In addition to regulating its primary target, glycogen synthase, GSK3β is involved in other fundamental cellular pathways depending on its substrates and binding partners [66-68]. GSK3β is a potential therapeutic target for common chronic diseases including type 2 DM and Alzheimer's disease, given the causative associations with glucose intolerance, neurodegenerative disorders, and inflammation [69-71].

### 5.2. Pathologic Role of GSK3β in Various Cancer Types

Under physiologic conditions, GSK3β phosphorylates several transcription factors (e.g., c-Jun, c-Myc), cell cycle regulators (e.g., cyclin D1), and proto-oncoproteins (e.g., β-catenin), thereby triggering their degradation via the ubiquitin-proteasome system. GSK3β is, therefore, hypothesized to inhibit tumor development by interfering with oncogenic signaling (e.g., Wnt, hedgehog) [72]. However, there is little evidence that links GSK3β inactivation or loss of GSK3β expression with tumor development.

In the last five years, we demonstrated that deregulated expression, phosphorylation of S9 and Y216, and GSK3β activity are distinct features of gastrointestinal cancers including pancreatic cancers and glioblastoma, and that GSK3β sustains the survival and proliferation of these tumor cells. A pathologic role for GSK3β is supported by observations that inhibition of its activity reduced the survival and proliferation of different cancer cell types, predisposing them to apoptosis both *in vitro* and in tumor xenografts [73-76]. We also found that GSK3β inhibition in cancer cells was accompanied by restoration of p53 and Rb tumor suppressor pathways [75,76] and downregulation of human telomerase reverse transcriptase (hTERT), resulting in cell senescence [76]. This led us to propose GSK3β as a potential target for cancer treatment and to apply for domestic and international patents [77].

**Figure 3.**
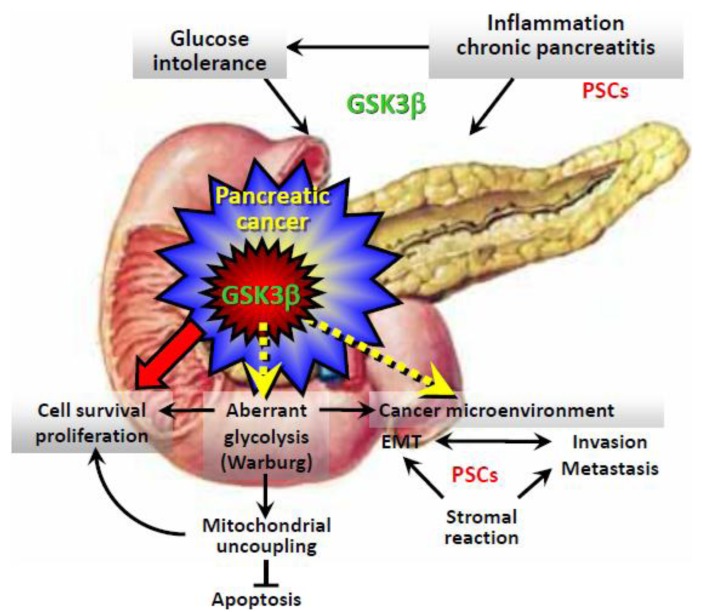
Systemic and local effects of aberrant GSK3β on risk factors (glucose intolerance and chronic inflammation) and progression of pancreatic cancer. Molecular mechanisms leading to the pathways indicated by the dotted arrows are not well characterized.

Simultaneously and following our studies on the antitumor effects of GSK3β inhibition, similar observations from other laboratories were reported for various cancer types with underlying mechanisms that included regulation by GSK3β inhibition of several pathways mediated by p53, Rb, p27^Kip1^, cyclin-dependent kinase (CDK), cyclin D1, c-Myc, and NF-κB [78]. Although the putative role of GSK3β in cancer is still debated [79,80], the overall results indicate that aberrant expression and activity of GSK3β is likely to be a common and fundamental characteristic of a broad spectrum of cancers (Figure 3) [78].

Based on the potential involvement of GSK3β in NF-κB-mediated cell survival [81,82], a number of studies demonstrated that GSK3β is involved in pancreatic cancer cell survival via the NF-κB pathway [83-85]. Few studies had focused on the role of GSK3β in the cellular response to chemotherapy until we reported that GSK3β inhibition sensitizes glioblastoma cells to chemotherapeutic agents (e.g., temozolomide, ACNU) and ionizing radiation [75]. However, a recent study failed to demonstrate that disrupting NF-κB activity by inhibiting GSK3β sensitizes PANC-1 pancreatic cancer cells to gemcitabine [85]. We previously found that GSK3β inhibition did not affect endogenous NF-κB transcriptional activity in tumor cells established from pancreatic cancers and glioblastoma [75,76]. In a preliminary study, we found that a small-molecule GSK3β inhibitor increased pancreatic cancer cell sensitivity to gemcitabine in cell culture and tumor xenografts when its dose and treatment protocol were optimized, and have identified the molecular mechanisms underlying the increased sensitivity [86]. Our findings indicate that GSK3β inhibition combined with chemotherapy is a novel and promising strategy to sensitize pancreatic cancer cells to gemcitabine.

### 5.3. Putative Antitumor Effects of GSK3β Inhibition via Modulation of Tumor Biology

Increasing evidence suggests that GSK3β participates in a wide range of physiological processes that determine cell fate, including cell motility, energy metabolism, and transcriptional control (reviewed in [78]). In addition to the pathologic roles of GSK3β in cancer cell survival and proliferation [73-78,83-85] as discussed above, GSK3β may influence critical biological properties of cancer cells, such as their dependence on glycolysis and invasive ability associated with EMT (Figure 3). Modulation of these biological properties by pharmacologic inhibition of GSK3β may sensitize pancreatic cancer cells to standard chemotherapy and radiation. Given the systemic pathology caused by aberrant GSK3β activity in glucose intolerance and chronic inflammation [71], GSK3β inhibition may decrease the risk of developing pancreatic cancer by improving these conditions. Accordingly, investigating the functions and pathologic roles of GSK3β should establish a firm molecular basis for future cancer treatments that target this kinase.

## 6. Perspectives

Here, we reviewed recent studies on the epidemiologic characteristics of pancreatic cancer and the molecular and biological mechanisms contributing to its development and progression, and highlight advances in alternatives to conventional treatments. Although molecular target-directed therapy is currently attracting considerable attention, especially for cancer refractory to standard chemotherapy and radiation, this therapy produces therapeutic effects that are still far from sufficient for most patients with advanced and recurrent pancreatic cancer. For many cancer patients, including those with pancreatic cancer, resistance to currently available therapeutics presents a major obstacle and is due to the aberrant metabolism of cancer cells, their microenvironment, their ability to invade and metastasize, and the acquisition of gene mutations. Multidisciplinary approaches directed to a more complete understanding of pancreatic cancer pathogenesis hold great promise in improving the outlook of this disease.

## Abbreviations

AMPK: 5′-AMP-activated protein kinase
ATP: adenosine triphosphate
CDK: cyclin-dependent kinase
COX-2: cyclooxygenase-2
CT: computed tomography
DCA: dichloroacetate
2-DG: 2-deoxy-D-glucose
DM: diabetes mellitus
DPC: deleted in pancreatic carcinoma
EGF: epidermal growth factor
EGFR: EGF receptor
EMT: epithelial-mesenchymal transition
FDG: [^18^F] fluoro-2-D-deoxyglucose
FGF: fibroblast growth factor
GSK3β: glycogen synthase kinase-3β
HGF: hepatocyte growth factor
hTERT: human telomerase reverse transcriptase
IGF-1: insulin-like growth factor-1
LKB1: liver kinase B1
MAPK: mitogen-activated protein kinase
mTOR: mammalian target of rapamycin
NADPH: nicotinamide adenine dinucleotide phosphate
NF-κB: nuclear factor-κB
NO: nitric oxide
iNOS: inducible NO synthase
PDGF: platelet-derived growth factor
PDGFR: PDGF receptor
PDH: pyruvate dehydrogenase
PDK1: PDH kinase 1
PET: positron emission tomography
PI3K: phosphatidylinositol 3′-kinase
PPARγ: peroxisome proliferator-activated receptor-γ
PSC: pancreatic stellate cell
ROS: reactive oxygen species
TCA: tricarboxylic acid
TGF: transforming growth factor
VEGF: vascular endothelial growth factor
VEGFR: VEGF receptor
